# Highly Dynamic Polynuclear Metal Cluster Revealed in a Single Metallothionein Molecule

**DOI:** 10.34133/2021/9756945

**Published:** 2021-07-14

**Authors:** Guodong Yuan, Felipe Curtolo, Yibing Deng, Tao Wu, Fang Tian, Qun Ma, Yutong Liu, Jinglin Zuo, Guilherme Menegon Arantes, Peng Zheng

**Affiliations:** ^1^State Key Laboratory of Coordination Chemistry, Chemistry and Biomedicine Innovation Center (ChemBIC), School of Chemistry and Chemical Engineering, Nanjing University, Nanjing, Jiangsu 210023, China; ^2^Department of Biochemistry, Instituto de Química, Universidade de São Paulo, Av. Prof. Lineu Prestes 748, 05508-900 São Paulo, SP, Brazil

## Abstract

Human metallothionein (MT) is a small-size yet efficient metal-binding protein, playing an essential role in metal homeostasis and heavy metal detoxification. MT contains two domains, each forming a polynuclear metal cluster with an exquisite hexatomic ring structure. The apoprotein is intrinsically disordered, which may strongly influence the clusters and the metal-thiolate (M-S) bonds, leading to a highly dynamic structure. However, these features are challenging to identify due to the transient nature of these species. The individual signal from dynamic conformations with different states of the cluster and M-S bond will be averaged and blurred in classic ensemble measurement. To circumvent these problems, we combined a single-molecule approach and multiscale molecular simulations to investigate the rupture mechanism and chemical stability of the metal cluster by a single MT molecule, focusing on the Zn_4_S_11_ cluster in the *α* domain upon unfolding. Unusual multiple unfolding pathways and intermediates are observed for both domains, corresponding to different combinations of M-S bond rupture. None of the pathways is clearly preferred suggesting that unfolding proceeds from the distribution of protein conformational substates with similar M-S bond strengths. Simulations indicate that the metal cluster may rearrange, forming and breaking metal-thiolate bonds even when MT is folded independently of large protein backbone reconfiguration. Thus, a highly dynamic polynuclear metal cluster with multiple conformational states is revealed in MT, responsible for the binding promiscuity and diverse cellular functions of this metal-carrier protein.

## 1. Introduction

Transition metals are essential ingredients for life and in a large fraction are covalently bound to proteins [1]. Typically, metals form stable metal-ligand coordination bonds as a protein metal cofactor or bound cluster, providing crucial catalytical or structural functions [2]. In addition, the protein secondary structure modifies the properties of the cluster and metal-ligand bonds [3]. For example, recent studies on nitrogenase showed that metal-coordinating sulfur atoms in the metal cluster could move during the enzymatic reaction, as a dynamic metallocofactor critical for catalysis, indicating how dynamic a metal cluster could be in a protein [4].

Metallothionein (MT) is another remarkable metalloprotein. It is an efficient metal-chelator protein with twenty metal-coordinating cysteines, capable of promiscuous binding more than ten different types of metals, forming two polynuclear metal clusters, M_4_S_11_ and M_3_S_9_, in its two domains, respectively [5, 6]. It is well known that Apo-MT is intrinsically disordered and highly flexible, which may strongly influence the clusters and lead to a dynamic structure with unique metal-thiolate (M-S) bonds. However, these features are challenging to identify due to the transient nature of the involved species. The individual signal from dynamic conformations of a protein with various populated states of the cluster and arrangements of M-S bonds will be averaged and blurred in classic ensemble characterization.

To circumvent these problems, we combined a single-molecule approach and multiscale molecular simulations to investigate the dissociation mechanism and chemical stability of the polynuclear metal cluster in a single human MT-III molecule, focusing on the Zn_4_S_11_ cluster in the *α* domain upon protein unfolding. Atomic force microscopy (AFM) is an advanced instrument in nanotechnology [7]. Using its ultrasharp AFM tip, it is able not only to image nanoscale surface but also to manipulate a single molecule mechanically as single-molecule force spectroscopy (SMFS) [8–12]. By stretching one molecule under defined coordination, SMFS can induce the conformational change of a molecule such as protein (un)folding [13–15], breaking a stable chemical bond as well as capturing important transient states [16–21]. For example, several metal-binding proteins have been studied by AFM-SMFS, in which the metal cluster is ruptured during protein unfolding [22–29]. Here, by using AFM-SMFS to study one MT molecule at a time, the dynamic nature of the polynuclear metal cluster in MT is revealed. Moreover, multiscale molecular simulations are performed to identify the M-S bonds broken upon mechanical stress and to investigate the intrinsic stability of the exquisite M_4_S_11_ cluster in the folded *α*MT. Altogether, a detailed picture of cluster reconfiguration and M-S bond rupture for human MT-III is revealed.

**Figure 1:**
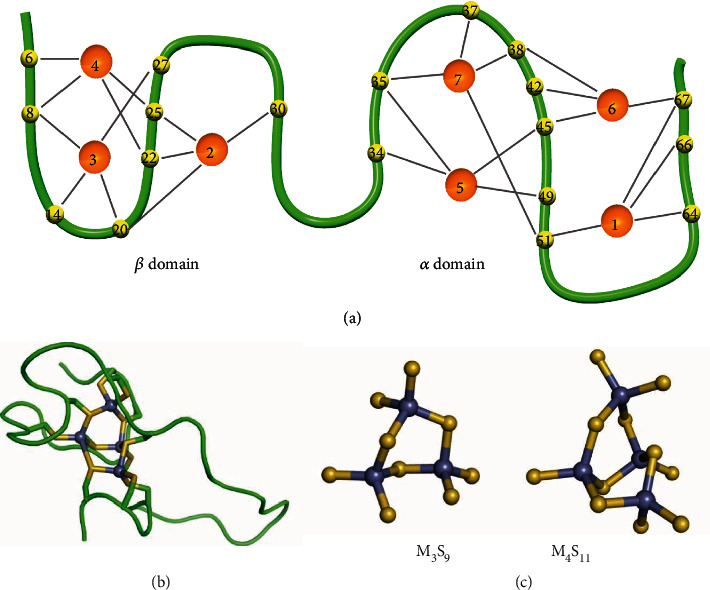
Structure of metallothionein and its bound polynuclear metal clusters. (a) Human MT-III schematics showing the N-terminal *β*-domain and C-terminal *α*-domain. The metals (in orange) and cysteines (in yellow) along the protein backbone (in green) are numbered based on previous studies [9, 17]. (b) Folded *α*-domain of MT-III with the protein backbone and cysteine side chains, with sulfur in yellow and cadmium ions in gray (PDB 2F5H) [15]. (c) Hexatomic ring structure of the M_3_S_9_ cluster (left) found in *β*MT and M_4_S_11_ cluster (right) in *α*MT.

Considerable efforts have gone into the study of the folding mechanism of MT upon metal addition, indicating partially metallated intermediate transition from extended to more compact conformations [30, 31], a clear example of folding induced by metal binding. Solution NMR has been able to reveal an ordered and folded structure for both N-terminal *β* (residues 1-31, MT-III numbering) and C-terminal *α* (residues 32-68) domains in a fully metallated form (Figure 1(a)) [31–35].

In *α*MT, 11 cysteines bind to 4 metal ions to form the M_4_S_11_ cluster (Figures 1(b) and 1(c)) in a canonical arrangement of two 6-membered (hexatomic) rings fused in two edges, with 5-bridge (bound to two metals) and 6-terminal (bound to one metal) sulfurs. In *β*MT, one hexatomic ring forms the M_3_S_9_ cluster (Figure 1(c)). All metal centers in these clusters show slightly distorted tetrahedral coordination in the more than 40 MT structures available from several organisms [34, 36]. For divalent cations, there are only two reports suggesting alternative metal-thiolate (M-S) connectivity, with trigonal coordination and short metal-metal distances attributed to dynamic cluster rearrangements. However, the signal for a specific M-S bond and cluster configuration is blurred in classic ensemble methods that average upon multiple cluster connectivities, protein conformations, and a possibly heterogeneous MT sample as mentioned [35].

## 2. Results and Discussion

### 2.1. One-Step Rupture of Each Metal Cluster upon MT Unfolding

**Figure 2:**
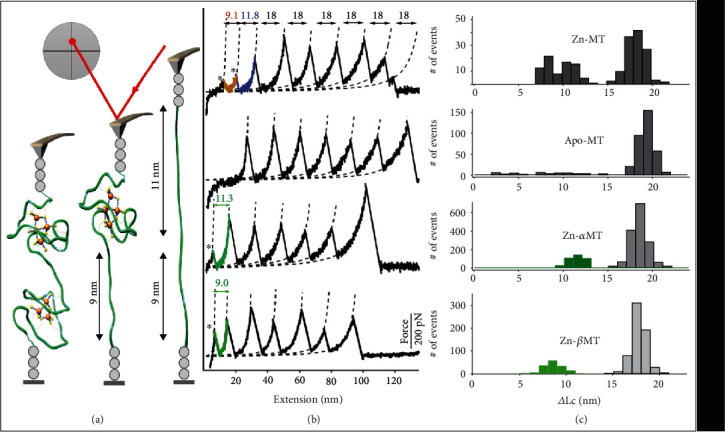
Characterization of single MT molecule by AFM-SMFS unfolding experiments. (a) Scheme of the AFM experiment shows the unfolding events of the two domains in MT. The unfolding of six GB1 (gray circle) at last is omitted for simplicity. (b) Representative force-extension unfolding curves of different forms of Zn-MT polyprotein from the one-step unfolding of *α*MT with ΔLc of 11 nm, *β*MT with ΔLc of 9 nm, and GB1 with ΔLc of 18 nm (curve 1 for Zn-MT, 2 for Apo-MT, 3 for Zn-*α*MT, and 4 for Zn-*β*MT. (c) ΔLc histogram of the corresponding protein construct indicates the three distributions for the one-step unfolding of *α*MT and *β*MT. The bin size is 1 nm. Raw data for (c) is provided as a source data file.

The characterization of a single MT molecule was achieved by single-molecule AFM unfolding experiments. Polyprotein (GB1)_3_-MT-(GB1)_3_ is designed to ensure detection of single-molecule events, in which target MT is sandwiched by previously characterized marker protein GB1s (contour length increase of 18 nm and force of 180 pN upon unfolding) (Figure 2, Figures S1–S3, and Supplementary Note 2) [37, 38]. A proper amount of polyprotein solution is evenly deposited on the glass coverslip to optimize the single-molecule pickup ratio and reliability. Then, the AFM cantilever presses onto the coverslip and captures one molecule. By moving the cantilever vertically, a polyprotein is stretched from two ends and unfolded, leading to the rupture of M-S bonds and disruption of the MT metal cluster (Figure 2(a)). By repeating this cycle 10^5^ times, thousands of single MT mechanical unfolding trajectories were measured.

Wild-type MT-III bound to Zn (Zn-MT) was studied first. Stretching the polyprotein by AFM-SMFS resulted in sawtooth-like force-extension curves with multiple peaks, as shown in Figure 2(b). These peaks are from the unfolding event of the MT or GB1. Besides the multiple GB1 unfolding events with ΔLc of 18 nm (colored in black), we found two peaks (Figure 2(b), curve 1, colored in brown and purple) with contour length increment (ΔLc) that correspond to MT unfolding events: a peak with ΔLc of 11.6 ± 0.9 nm (ave.±stdv., *n* = 43) and another one with ΔLc of 8.6 ± 0.7 nm (*n* = 43) (Figure 2(c)) [39]. The 11 nm peak corresponds to the unfolding of the MT *α*-domain and rupture of its Zn_4_S_11_ cluster, which leads to an extension of 34 amino acids (aa) between the first Cys34 (bound to Zn5) and the last Cys67 (bound to Zn6, Figure 1(a)). Such value can be calculated from 0.36 nm/aa∗34 aa − 1.3 nm = 10.9 nm (0.36 nm is the length increment of one aa, and 1.3 nm is the distance between Cys34 and Cys67 in the folded MT-III, PDB 2F5H) [40, 41]. Similarly, unfolding of the *β*-domain and rupture of the Zn_3_S_9_ cluster lead to an extension of 25 aa between Cys6 (bound to Zn4) and Cys30 (bound to Zn2) and a calculated ΔLc of 7.8 nm (0.36 nm/aa∗25 aa − 1.2 nm, taken from the *β*MT-II *β*-domain as there is no structure for *β*MT-III available). Measured and calculated contour length increments agree reasonably [40].

Here, the polynuclear clusters behave as entire structural units that rupture and lead to a single force peak upon MT unfolding (Figure 2(b)), confirmed by several control experiments (Figures 2(b) and 2(c)). Apo-MT prepared without both metal clusters showed neither 9 or 11 nm peaks (curve 2, Figure 2(b)). Truncated MT polyprotein to only *α*- or *β*-domain showed the 11 nm peak (curve 3, 11.3 ± 0.9 nm, *n* = 418) or 9 nm peak (curve 4, 8.8 ± 1.0 nm, *n* = 125), respectively, and these peaks disappeared in the corresponding Apo forms (Figure S4). Experiments on Apo-MT verified that protein secondary structure does not contribute significantly to the force necessary to unfold MT domains so that the metal cluster is the primary determinant of MT structure and stability, in agreement with previously ensemble experiments [31, 32].

### 2.2. Multiple Two-Step Ruptures of the Zn_4_S_11_ Cluster in *α*MT

**Figure 3:**
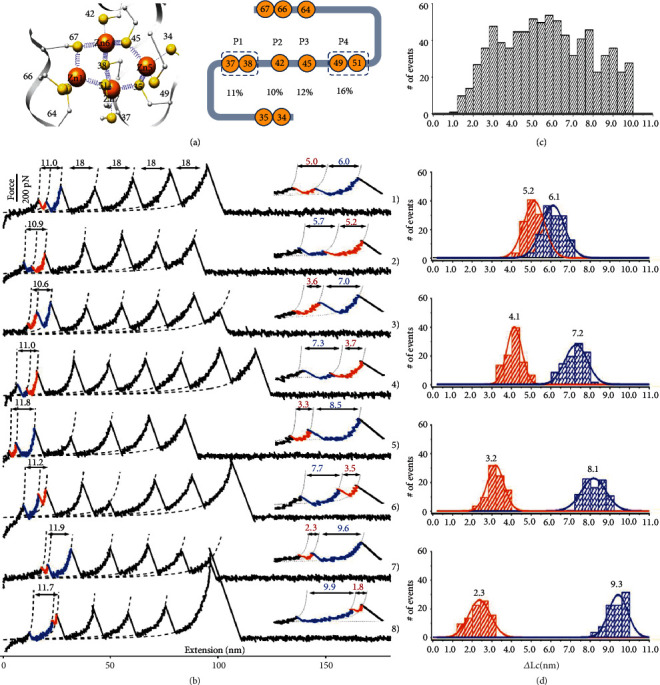
The multiple two-step rupture scenario of the Zn_4_S_11_ cluster in *α*MT detected by single-molecule AFM. (a) Scheme of *α*MT metal-binding cysteines. (b) Representative curves of the four different two-step rupture pathways of *α*MT. The two peaks from the rupture of the Zn_4_S_11_ cluster are fitted by a dashed line and enlarged for clarity. The sum of their ΔLc value (11-12 nm) is written in green. (c) ΔLc histogram of all two-step ruptures of *α*MT shows a broad and continuous distribution from 1 to 10 nm. The bin size is 0.4 nm. (d) ΔLc histogram of *α*MT for each rupture pathway corresponding to curves shown in (b). Raw data for (c) and (d) are provided as a source data file.

Besides the one-step unfolding events, we also observed a stepwise unfolding scenario for the *α*-domain with two consecutive peaks (*n* = 406, Figure 3(b)) resolved using a strict data selection criterion (Supplementary Note 2). Remarkably, the ΔLc of these two consecutive peaks always added up to ~11 nm but with a broad and continuous distribution ranging from 1 to 10 nm (Figures 3(c) and 3(d), Figure S5). In fact, we also detected stepwise unfolding for both domains in the full-length MT (Figure S6). However, their similar ΔLc signals hampered a clear identification and characterization of each cluster, so we studied each domain separately (Supplementary Note 3).

While one-step unfolding of *α*MT proceeds by disruption of the entire polynuclear metal cluster and leads to an extension of 34 residues enclosing the cluster (segment Cys34-Cys67, Figure 3(a)), the two-step events require that an unfolding intermediate is formed with at least one anchoring cysteine in the middle of this segment (Cys37 (or Cys38), Cys42, Cys45, and Cys49 (or Cys51)) still connected to the metal centers. Due to the length resolution in the AFM-SMFS experiment of about three amino acids (~1 nm), Cys51 cannot be distinguished from Cys49 nor Cys38 from Cys37.

In Table 1, we deconvolute the measured ΔLc histogram (Figure 3(c)) into four combinations or pathways (P1-P4), each corresponding to the maintenance of one anchoring cysteine bound in the unfolding intermediate, based on its theoretical ΔLc (Table S1, Supplementary Note 4): 5.2 + 6.1 nm (P1, *n* = 132) with Cys49 (or Cys51) bound, 4.1 + 7.2 nm (P2, *n* = 98) with Cys45 bound, 3.2 + 8.1 nm (P3, *n* = 86) with Cys42 bound, and 2.3 + 9.3 nm (P4, *n* = 90) with Cys37 (or Cys38) bound (Figures 3(a) and 3(d), Figure S7).

For instance, the ΔLc of two-colored peaks in curves 3 and 4 of Figure 3(b) adds up to ~11 nm, indicating they were from the two-step rupture of *α*MT. In curve 3, the first peak was fitted to ΔLc = 3.6 nm, which agrees with the extension of segment Cys34-Cys45 (12 aa, calculated ΔLc = 3.6 nm, Table S1) when unfolding initiates from the N-terminus. The second peak has ΔLc = 7.0 nm, which agrees with the unfolding of the remaining Cys45-Cys67 segment (22 aa, calculated ΔLc = 7.5 nm, Table S1). Thus, this curve belongs to pathway P2 (average ΔLc = 4.1 + 7.2 nm, Table 1) with Cys45 still bound to the metal cluster in the unfolding intermediate (Figure S7). Curves showing force peaks with similar values but in reverse order were also detected, such as curve 4, with a 7.3 nm peak followed by a 3.7 nm peak. This indicates that *α*MT can unfold from both ends, and unfolding events starting from the C-terminus may also have an intermediate with an anchoring cysteine (Cys45 in this example) still bound to the metal cluster. More representative curves are shown in supporting information (Figure S8).

It is fortunate that a long cysteine-free fragment between residues 51 and 64 with ΔLc upon the unfolding of ~4.5 nm is present in *α*MT (Figure 3(a)). This enabled us to identify the unfolding direction as the peak with a larger ΔLc must come from the C-terminus. From all measured two-step trajectories (*n* = 406), we found that 55% unfold from the C-terminus and 45% from the other terminus (Figure S6).

The eight two-step rupture scenarios, corresponding to four pathways in two possible directions, were observed with similar frequencies (Table 1). This indicates the presence of at least 8 unfolding intermediates. The M-S bonds in the cluster have similar strengths whose rupture is stochastically induced by fluctuations of the flexible protein backbone. These observations also suggest that the *α*MT structure has static disorder and is better represented by a configurational distribution, such as in a molten protein globule and in line with the intrinsically disordered nature of Apo-MT [34, 35]. The M-S bond pattern and metal cluster configuration should also exchange in response to protein conformations. When the stability of the metal cluster is probed one molecule at a time (as in AFM-SMFS), a distribution of unfolding pathways and intermediates is observed. In fact, the real number of the two-step rupture pathways and intermediates of *α*MT under mechanical unfolding is most likely more than eight. Unfolding events assigned to pathway 1 or 4 may include Cys51 or Cys38 as anchoring cysteine, which would lead to other intermediates. Moreover, we observed few *α*MT unfolding curves with more than two steps (<1%, Figure S9). Altogether, these results indicate that the Zn_4_S_11_ metal cluster in *α*MT is highly plastic and dynamic.

Cadmium-substituted *α*MT (Cd-*α*MT) was also investigated, and two-step rupture scenarios were detected, again with a wide and continuous ΔLc distribution (Figure S10). Comparable frequencies of one- and two-step curves were observed (Table 1), and the same four pathways could be assigned, as found for Zn-*α*MT, indicating that the unfolding mechanism of the M_4_S_11_ cluster and the stability of M-S bonds are mainly determined by the protein sequence and cluster topology, but not by the nature of the bound metal. AFM-SMFS measurements also show that Zn-*α*MT has a lower rupture force of 75 ± 34 pN (for one-step ruptures) than Cd-*α*MT which has a force of 120 ± 61 pN (Figure S11). This result agrees with ensemble measurements that Cd-*α*MT is more stable and that free Cd^2+^ can replace zinc in Zn-MT [35].

Finally, we performed AFM-SMFS experiments on Zn-*β*MT using the (GB1)_3_-*β*MT-(GB1)_3_ construct. Again, one- and two-step unfolding events of *β*MT with a broad and continuous ΔLc distribution (from 1 to 9 nm, *n* = 82) were observed (Figure S12), suggesting that M-S bonds in *β*MT also have similar property and that multiple mechanical unfolding pathways and intermediates are possible. However, we are unable to determine unfolding direction or anchoring cysteine because many of these residues are adjacent in the *β*MT sequence and impossible to resolve by the AFM measurement (Supplementary Note 5). Nevertheless, the similar multiple unfolding pathways and a large fraction of two-step unfolding events indicate that the Zn_3_S_9_ cluster in the *β* domain is also highly dynamic like the *α* domain.

### 2.3. Molecular Modeling Confirms a Distribution of Unfolding Pathways and a Plastic Metal Cluster

**Figure 4:**
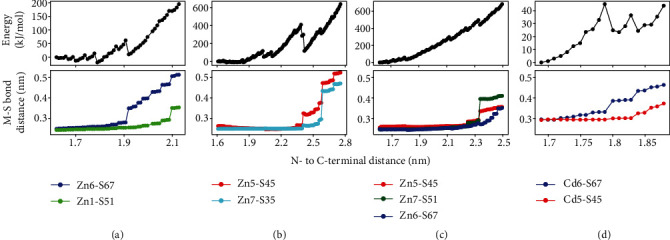
Mechanical unfolding profiles for *α*MT obtained from molecular simulations. Upper panels show the total potential energy, and lower panels show the distance of broken metal-thiolate bonds (M-S, as colored in the legend) upon increasing the protein N- and C-terminal distance. (a) to (c) were obtained for Zn-*α*MT with starting geometries, respectively, taken from 1 ns, 3 ns, and 5 ns snapshots of the initial classical molecular dynamic simulations, and (d) was obtained for Cd-*α*MT from the 5 ns snapshot (the same as in (c)). Raw data for this figure is provided as a source data file.

To identify the M-S bonds broken upon mechanical stress, we employed molecular modeling and simulated the forced unfolding of the *α*MT protein using a hybrid QM/MM potential and the constrained geometry emulating force procedure (COGEF, see Supplementary Methods for details) [42–46]. These simulations employ a quantum-chemical model and naturally describe the formation and rupture of M-S bonds. Upon stretching the protein termini, equivalent to the AFM-SMFS experiment, the energy of the full system increases as intramolecular contacts are stressed and the protein structure is destabilized. Spikes followed by energy drops correspond to disruption of some of these interactions and subsequent structural relaxation. Small spikes (e.g., Figure 4(a), 1.6-1.8 nm terminal distance) represent the disruption of H-bonds and electrostatic contacts within the protein. Larger spikes (with higher energy drop) correspond to the M-S bond rupture (lower panels in Figure 4). Zn-S distances higher than 0.30 nm and Cd-S distances higher than 0.33 nm indicate broken bonds, as the Cd-S equilibrium bond distance is higher. We carried on the simulations until at least two M-S bonds were broken.

In the representative profiles shown in Figure 4, the same 16 M-S bonds were initially present and form the *α*MT metal cluster in its canonical shape (Figure 1(c)). Upon stress, different M-S bonds are broken (a total of 5 in the examples shown). All of which are bridge thiolates, suggesting that these are weaker bonds due to the cluster topology and dual-metal bonding. Figures 4(a)–4(c) indicate that mechanical unfolding of a slightly different initial protein configuration, sampled by short (ns time scale) classical molecular dynamic simulations, led to the rupture of different M-S bonds, in agreement with the interpretation of AFM-SMFS experiments. For instance, the rupture of Zn6-S67 and Zn1-S51 in Figure 4(a) will unfold the *α*MT C-terminal and lead to a ΔLc of ~6 nm as in the orange peaks of curve 1 in Figure 3(b) (P1 in Table 1). Simulations exchanging Zn for Cd, but with the same protein conformation (Figures 4(c) and 4(d)), indicate that similar M-S bonds rupture (M6-S67 and M5-S45), although the Zn cluster is more labile and an extra M-S bond ruptures (Zn7-S51). Thus, the type of bound metal has a small influence on M-S bond rupture and consequently the MT unfolding pathway, in line with the AFM-SMFS results for *α*MT-III.

**Figure 5:**
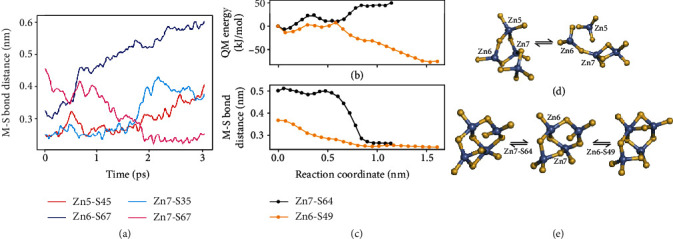
Plasticity of the polynuclear metal cluster in folded *α*MT. (a) Time evolution of four M-S distances resulting in spontaneous cluster reconfiguration along a molecular dynamic simulation (without any mechanical or external forces applied), with initial and final cluster geometries shown in (d). The color code shown in the legend corresponds to Figure 4 and SI Video. (b, c) Quantum mechanical (QM) energy and M-S distances for two additional reactions starting from the cluster geometry shown in the middle of (e) and leading to the formation of the Zn7-S64 bond (corresponding to black curves in (b) and (c)) or the Zn6-S49 bond (orange curves) as shown in the left and right geometries of (e), respectively. Raw data for this figure is provided as a source data file.

We also investigated the stability of the metal cluster in *α*MT without any mechanical stress by carrying out simulations of the folded Zn-*α*MT domain with the hybrid QM/MM potential without any restraints or external forces [44, 47, 48].  Figure 5(a) shows the evolution of four M-S distances illustrating the reconfiguration of the metal cluster in folded *α*MT (Figure 5(d) and SI Video). When zinc ion is bound to *α*MT in the original configuration (PDB 2F5H) [32], one M-S bond is lost (Zn6-S67 distance is higher than 0.30 nm, Figure 5(a)) and 15 Zn-S bonds are formed in comparison to the canonical 16 Cd-S bonds (Figure 1(c)). This is due to the smaller Zn-S equilibrium bond length and accompanying cluster distortion. Following a short molecular dynamics trajectory (3 ps), we observe significant cluster reconfiguration with the formation of Zn7-S67 and rupture of Zn5-S45 and Zn7-S35 bonds. The final configuration shows only 14 M-S bonds and two metal centers (Zn5 and Zn6) with 3 M-S bonds in trigonal shape. In comparison, the protein backbone changes little during the trajectory (C*α* root mean-squared deviation of 0.09 nm). These spontaneous reactions suggest that the polynuclear metal cluster in folded *α*MT may change its coordination and shape fast independently of large fluctuations in the protein backbone.

Spontaneous addition reactions when Zn6 and Zn7 formed an extra (5th) M-S bond were also observed in other reactive MD trajectories, so the energetics of these two reactions were characterized as extra examples of cluster reconfiguration (Figures 5(b) and 5(c)). Starting from a canonical cluster geometry with 16 Zn-S bonds (middle of Figure 5(e), the same geometry as that in Figure 4(c)), the addition of Zn7-S64 or Zn6-S49 bonds was observed with rather low barriers, respectively, 52 and 21 kJ/mol (Figure 5(b)). The formation of the Zn6-S49 bond is stable, and the low barrier can be easily activated by thermal motion, so this cluster configuration should be often found for folded Zn-*α*MT in solution.

These results indicate that spontaneous cluster reconfiguration in folded *α*MT may start from either M-S bond rupture (as in Figures 5(a) and 5(d)) or addition (Figures 5(b), 5(c), and 5(e)). Although we have not carried out an exhaustive search of cluster reaction mechanisms nor sampling of protein configurations, the spontaneous reactions found here with low barriers and stable M-S bond arrangements different from the canonical form (Figure 1(c)) support the plasticity of the cluster in folded *α*MT and agree with the interpretation of the AFM-SMFS experiments above.

### 2.4. Protein and Cluster Topologies Determine *α*MT Unfolding Kinetics

**Figure 6:**
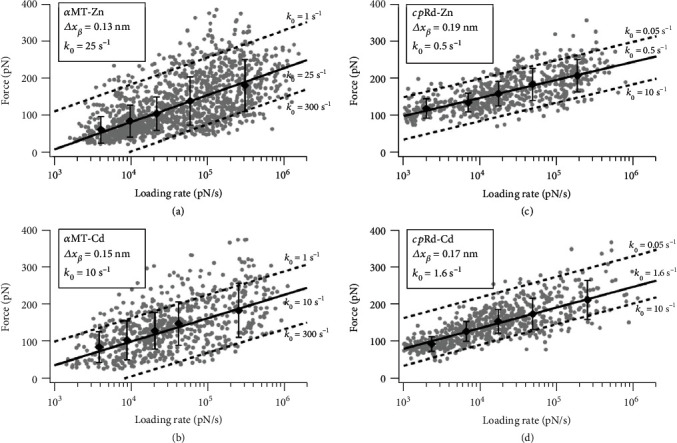
Comparison of unfolding kinetics for *α*MT with an M_4_S_11_ metal cluster and Rd with an MS_4_ metal center. Semilogarithmic representation of the rupture force of Zn-*α*MT (a), Cd-*α*MT (b), Zn-Rd (c), and Cd-Rd (d) as a function of the loading rate. Gray dots represent cluster break events, and black diamonds represent the most probable rupture force under five different force-loading rate intervals between 1000 and 300,000 pN s^−1^. The black line represents the linear fit to the five force values based on the Bell-Evans model. Fitted parameters Δ*x* and *k*_off_ are shown in legend boxes for each protein. The dashed line is a hypothetic fitting with a larger or smaller *k*_off_ value to indicate the accuracy of our fitting.

We also obtained the kinetics of Zn- and Cd-*α*MT unfolding in a one-step rupture scenario by AFM in a dynamic force spectroscopy mode (DFS). Based on the Bell-Evans model, the application of mechanical force on a chemical bond lowers its activation energy barrier towards the bond dissociation. It also describes that the force is proportional to the logarithm of the loading rate as observed here for the metal cluster rupture (Figures 6(a) and 6(b)) [49, 50]. As a result, spontaneous bond dissociation/break rate (*k*_off_) and distance from a bound state to dissociation transition state (Δ*x*) were obtained from the force vs. loading rate plot (Figure 6), with *k*_off_ = 25 s^−1^ for Zn-*α*MT and 10 s^−1^ for Cd-*α*MT. These *k*_off_ values are about ten times higher than the value of most metal-ligand bonds in protein systems. The higher *k*_off_ for Zn-*α*MT is also in line with its lower unfolding force and lower ensemble stability noted above.

To disentangle the contribution of metal-thiolate bonds from the protein and cluster configurations, we compared kinetic measurements with the rubredoxin (Rd), containing a simple Fe(Scys)_4_ center (a single metal ion with four thiolate bonds in tetrahedral coordination, Figure S11D) [51, 52]. Rubredoxin is a stable, compact, and globular protein involved only in electron transfer, which can also be prepared as Zn- and Cd-bound forms (see SI). Our AFM measurements in a dynamic mode showed that *k*_off_ is 0.5 s^−1^ for Zn-Rd and 1.6 s^−1^ for Cd-Rd (Figures 6(c) and 6(d)). Thus, the 50x faster rate of bond dissociation rate measured by a dynamic AFM mode in *α*MT is not intrinsic of the M-S bond but due to the unique polynuclear metal cluster bound to this protein.

## 3. Conclusion

We have combined a single-molecule approach and computational modeling to identify multiple unfolding pathways and characterize the chemical stability of bound polynuclear metal clusters in human MT-III protein. By studying one metallothionein molecule at a time, the previous hidden or averaged signals from individual MT with a dynamic cluster and metal-thiolate bond are revealed. AFM-SMFS unfolding results show that *α*- and *β*MT-III domains, respectively, bound to M_4_S_11_ and M_3_S_9_ metal clusters, where *M* = Zn^2+^ or Cd^2+^, unfold mostly noncooperatively. Both MT-III domains may unfold with (two-step) or without (one-step) intermediates. For *α*MT, we show that at least eight intermediates are possible for four different anchoring cysteines unfolding from N- or C-terminus. The number of intermediates is still a lower limit because the AFM-SMFS resolution cannot distinguish the unfolding of segments with adjacent cysteines. These results are unique among small proteins (less than 40 aa) studied with SMFS so far, which usually show a one-step unfolding process [53, 54]. Here, the hexametric metal cluster and its corresponding M-S bonds are responsible for multiple unfolding pathways.

Comparing Zn- to Cd-bound *α*MT, we find similar unfolding pathways and frequencies in AFM and M-S bonds ruptured in simulations. But Zn-*α*MT has a lower rupture force and slightly faster bond rupture kinetics, in line with ensemble measurements showing that Cd-*α*MT is more stable [35]. The rupture kinetics of Zn-S and Cd-S bonds in a mononuclear metal cluster scaffold (rubredoxin) is significantly slower than that in *α*MT, and the order between each metal is reversed. In fact, the rupture kinetics of Zn-*α*MT-III is the fastest observed to date for dissociation of chemical bonds in protein systems under mechanical stress. The lower stability of the Zn_4_S_11_ cluster is due to shorter Zn-S bonds, which perturbs the ring structure of the polynuclear cluster.

Modeling the *α*MT-III mechanical unfolding showed that at least 5 different M-S bonds might initially dissociate out of the 16 M-S bonds comprising the canonical M_4_S_11_ cluster. All ruptured bonds are formed by bridge cysteines, each with comparable stability but weaker than terminal cysteine bonds. Simulations also show that metal cluster rearrangement is possible in folded Zn-*α*MT-III, with M-S bond rupture, addition, and exchange that lead to stable cluster configurations different from the canonical form (Figure 1(c)) and uncoupled from large fluctuation in the protein backbone.

Depending on the metal type and protein sequence, MT metallation may proceed cooperatively or stepwise with the intermediate binding of up to 7 metal ions [33, 35, 55]. It performs fundamental cellular processes in humans such as metal homeostasis (Zn^2+^ and Cu^+^) and heavy metal detoxification (Cd^2+^ and Hg^2+^), both implicated in oxidative stress and neurodegenerative diseases. Despite the high MT-metal binding affinity and nuclearity, metallation kinetics is fast and happens in the millisecond scale, whereas the exchange of metal ions within folded domains or with solution takes place in minutes. Moreover, this high flexibility complicates MT structural characterization, as most structures obtained are from solution NMR. Our results of the highly plastic and dynamic nature of the polynuclear metal cluster in MT by AFM-SMFS and theoretical calculation agree well with the function of MT [42, 56, 57]. This dynamic nature renders MT as a thermodynamic stable but kinetically labile metal-binding protein, capable of efficient metal storage and release.

## Figures and Tables

**Table 1:** Statistics of different rupture pathways of the M_4_S_11_ cluster upon *α*MT unfolding.

Path	Measured value	Prob. of Zn-*α*MT	Prob. of Cd-*α*MT	Anchor cys
One step	11.3	51%, *n* = 418	52%, *n* = 188	—
P1	5.2 + 6.1/6.1 + 5.2	16%, *n* = 60/*n* = 72	17%, *n* = 32/*n* = 32	Cys49(51)
P2	4.1 + 7.2/7.2 + 4.1	12%, *n* = 44/*n* = 54	12%, *n* = 22/*n* = 21	Cys45
P3	3.2 + 8.1/8.1 + 3.2	10%, *n* = 39/*n* = 47	11%, *n* = 20/*n* = 20	Cys42
P4	2.4 + 9.3/9.3 + 2.4	11%, *n* = 40/*n* = 50	8%, *n* = 14/*n* = 15	Cys37(38)

## Data Availability

Data supporting the findings of this work are available within the paper and its supplementary information files. The datasets generated and analyzed during the current study are available from the corresponding author upon request. The data underlying Figures 2(c), 3(c), 3(d), 4, and 5 are provided as a source data file.

## Supplementary Materials

Supplementary Materials Supplementary Methods: (1) protein engineering, (2) protein characterization, (3) Apo-MT and Holo-MT preparation, (4) rubredoxin preparation, (5) UV absorbance of MT polyproteins, (6) single-molecule AFM unfolding experiment, (7) Bell-Evans model to extract the kinetic from loading rate experiment, and (8) molecular dynamics simulations and hybrid QM/MM calculations. Supplementary Notes: (1) the amino acid sequence of each protein. (2) AFM data selection criteria and data analysis procedure. (3) Why is single-domain MT used for metal cluster characterization? (4) The assignment of two-step unfolding events to four different pathways. (5) Boundary cysteine during the stepwise unfolding of *β*MT is difficult to resolve. Supplementary Figures. Figure S1: (A) SDS-PAGE gel results for MT polyproteins (GB1)3-xMT-(GB1)3. Figure S2: the MALDI-TOF-MS result of Zn-MT polyprotein (GB1)3-xMT-(GB1)3. Zn-MT (theoretical MW: 47.980 kD), Zn-*α*MT (44.673 kD), and Zn-*β*MT (43.938 kD). Figure S3: the UV-vis absorbance spectrum of MT polyprotein (GB1)3-xMT-(GB1)3. Cd-*α*MT, Zn-*α*MT, Zn-*β*MT, and Zn-MT. Figure S4: AFM unfolding results of Apo-*α*MT and Apo-*β*MT. Figure S5: ΔLc histogram of unfolding/rupture peaks in the two-step rupture scenarios of Zn-*α*MT (A) and Cd-*α*MT (B). Figure S6: force-extension curves of stepwise ruptured (GB1)3-MT-(GB1)3 showing peaks with similar ΔLc value from two domains. Figure S7: possible 2D schematics of the rupture mechanism of the M4S11 cluster. Figure S8: more representative unfolding curves of the four different two-step rupture pathways of the Zn4S11 cluster in *α*MT detected by AFM-SMFS, including 3.2 + 8.1 nm (A), 2.3 + 9.3 nm (B), 5.2 + 6.1 nm (C), and 4.1 + 7.2 nm (D), unfolding from both termini. Figure S9: force-extension curves of the multistep rupture scenario of the Zn4S11 in *α*MT. (A) Five multistep curves were found among ~800 *α*MT unfolding curves (<1%). Figure S10: AFM unfolding experiment results of Cd-*α*MT. Figure S11: rupture force histogram of one-step unfolding of Zn/Cd-*α*MT and Rd. Figure S12: AFM unfolding experiment results of Zn-*β*MT. Supplementary Table. Table S1. Theoretical ΔLc for the four two-step rupture scenarios of *α*MT. Supplementary Video: animation of the molecular dynamics simulation presented in Figure 5(a), using the same color code for M-S bonds. Distances in angstroms and two viewing angles are shown. The protein backbone and Cys side chains are in green tubes, with sulfur in yellow and zinc in gray.
