# Supplementary material for: Highly Dynamic Polynuclear Metal Cluster Revealed in a Single Metallothionein Molecule
Source: Research (Wash D C). 2021 Jul 14;2021:9756945. doi: 10.34133/2021/9756945 (PMC8299258; doi:10.34133/2021/9756945)
Supplement: Supplementary Materials — Supplementary Methods: (1) protein engineering, (2) protein characterization, (3) Apo-MT and Holo-MT preparation, (4) rubredoxin preparation, (5) UV absorbance of MT polyproteins, (6) single-molecule AFM unfolding experiment, (7) Bell-Evans model to extract the kinetic from loading rate experiment, and (8) molecular dynamics simulations and hybrid QM/MM calculations. Supplementary Notes: (1) the amino acid sequence of each protein. (2) AFM data selection criteria and data analysis procedure. (3) Why is single-domain MT used for metal cluster characterization? (4) The assignment of two-step unfolding events to four different pathways. (5) Boundary cysteine during the stepwise unfolding of βMT is difficult to resolve. Supplementary Figures. Figure S1: (A) SDS-PAGE gel results for MT polyproteins (GB1)3-xMT-(GB1)3. Figure S2: the MALDI-TOF-MS result of Zn-MT polyprotein (GB1)3-xMT-(GB1)3. Zn-MT (theoretical MW: 47.980 kD), Zn-αMT (44.673 kD), and Zn-βMT (43.938 kD). Figure S3: the UV-vis absorbance spectrum of MT polyprotein (GB1)3-xMT-(GB1)3. Cd-αMT, Zn-αMT, Zn-βMT, and Zn-MT. Figure S4: AFM unfolding results of Apo-αMT and Apo-βMT. Figure S5: ΔLc histogram of unfolding/rupture peaks in the two-step rupture scenarios of Zn-αMT (A) and Cd-αMT (B). Figure S6: force-extension curves of stepwise ruptured (GB1)3-MT-(GB1)3 showing peaks with similar ΔLc value from two domains. Figure S7: possible 2D schematics of the rupture mechanism of the M4S11 cluster. Figure S8: more representative unfolding curves of the four different two-step rupture pathways of the Zn4S11 cluster in αMT detected by AFM-SMFS, including 3.2 + 8.1 nm (A), 2.3 + 9.3 nm (B), 5.2 + 6.1 nm (C), and 4.1 + 7.2 nm (D), unfolding from both termini. Figure S9: force-extension curves of the multistep rupture scenario of the Zn4S11 in αMT. (A) Five multistep curves were found among ~800 αMT unfolding curves (<1%). Figure S10: AFM unfolding experiment results of Cd-αMT. Figure S11: rupture force histogram of one- [file 9756945.f1.zip › SI_MT4.docx]

Supplementary Information

Highly Dynamic Polynuclear Metal Cluster Revealed in a Single Metallothionein Molecule

Guodong Yuan^1#^, Felipe Curtolo^2#^, Yibing Deng^1#^, Tao Wu^1^, Fang Tian^1^, Qun Ma^1^, Yutong Liu^1^, Jinglin Zuo^1^, Guilherme Menegon Arantes^2^* and Peng Zheng^1^*

1：State Key Laboratory of Coordination Chemistry, Chemistry and Biomedicine Innovation Center, School of Chemistry and Chemical Engineering, Nanjing University, Nanjing, Jiangsu, 210023, China；2：Department of Biochemistry, Instituto de Química, Universidade de São Paulo, Av. Prof. Lineu Prestes 748, 05508-900, São Paulo, SP, Brazil;

# These authors contributed equally.

This Supplementary Information Includes:

**Supplementary Methods**

1. Protein engineering………………………………………………………..……. S3
2. Protein characterization……………………………………......…...…….…. S3-S4
3. Apo-MT and holo-MT preparation…………………….….………………..........S4
4. Rubredoxin preparation…………………………………….................................S4
5. UV-vis absorbance spectrum of the protein…….............................……….........S4
6. Single-molecule AFM unfolding experiment ………………….………….....S4-S5
7. Bell-Evans model to extract kinetic from loading rate experiment.......................S5
8. Molecular dynamics simulation.......................................................................S5-S6

**Supplementary Notes**

1. The amino acid sequence of proteins................................................................S7-S8
2. AFM data selection criteria and analysis procedure.........................................S8-S9
3. Why is single domain MT used for metal cluster characterization? .....................S9
4. The assignment of two-step unfolding events to four different pathways.............S9
5. Boundary cysteine in the stepwise unfolding of βMT is difficult to resolve...S9-S10

**Supplementary Figures**

Figure S1. SDS-PAGE gel results and AFM imaging for MT polyprotein.................S10

Figure S2. The MALDI-TOF-MS of Zn-MT polyproteins............................................S10

Figure S3. The UV absorbance spectrum of MT polyproteins......................................S11

Figure S4. AFM unfolding results of Apo-αMT and Apo-βMT...................................S11

Figure S5. ΔLc histogram of the unfolding of Zn/Cd (GB1)_3_-αMT-(GB1)_3_... ...........S12

Figure S6. Force-extension curves of stepwise ruptured (GB1)_3_-MT-(GB1)_3_..............S13

Figure S7. Possible 2D schematics of the rupture mechanism of the M_4_S_11_ cluster...S14

Figure S8. More representative curves of two-step unfolding event of Zn-αMT.......S15

Figure S9. Multistep unfolding curves of the Zn-αMT.................................................S16

Figure S10. AFM unfolding experiment results of Cd-αMT........................................S17

Figure S11. Rupture force histogram of Zn/Cd-αMT and Rd......................................S18

Figure S12. AFM unfolding experiment results of Zn-βMT.........................................S19

**Supplementary Table**

Table S1. Theoretical ΔLc for possible two-step rupture scenario of αMT.................S20

**Supplementary Video**...................................................................................................S20

**Supplementary References**................................................................................S20-S21

**Supplementary Methods**

**1. Protein engineering.** Human metallothionein III (MT) was used in this study. The genes encoding for protein MT, GB1, and Rd were ordered from Genscript Inc. The genes of polyprotein, (GB1)_3_-x(MT)-(GB1)_3_ and Cys-GB1-Rd-Cys were constructed in the expression vector pQE80L or pET32a using standard molecular biology techniques, where the GB1 (the B1 immunoglobulin-binding domain of *streptococcal* protein G, PDB:1PGA) was used as a protein marker for single-molecule detection. The procedures of protein expression, purification, and metal addition/depletion using Schlenk techniques are adopted from published references. MT proteins were overexpressed in *Escherichia coli* strain BL21(DE3) with the classic IPTG induction method using LB medium and M9 minimal medium supplemented with zinc salt. After sonication, the crude protein solution was purified by Co-affinity chromatography using TALON resins (Clontech.). The metal ion Zn or Cd was added by Schlenk techniques, described in detail in the following metal addition section. Further protein purification was performed using the gel filtration column Superdex 200 increase and the ion exchange column Mono Q5/50 using an FPLC machine (GE healthcare). The Cd-form protein was eluted according to the UV absorption at 250 nm generated from the characteristic absorption of the Cd-S bond. The polyprotein (GB1)_3_-MT-(GB1)_3_ was eluted at ~200 mM NaCl, and (GB1)_3_-αMT-(GB1)_3_ and (GB1)_3_-βMT-(GB1)_3_ were eluted at ~150 and ~140 mM.

**2. Protein characterization.** The polyproteins designed with full-length metallothionein or subdomain for single-molecule AFM unfolding experiments, (GB1)_3_-MT-(GB1)_3_, (GB1)_3_-αMT-(GB1)_3_ and (GB1)_3_-βMT-(GB1)_3_ were characterized by SDS-PAGE gel, AFM imaging and mass spectroscopy showing expected molecular weight as ~48 kD, ~45 kD, and ~44 kD, respectively (Fig. S1-S2). The mass spectroscopy measurements were performed using an Ultraflextreme MALDI-TOF/TOF machine from Bruker Corp.

To determine the concentration of Cd-αMT, its extinction coefficient of 45000 M^-1^cm^-1^ at 250 nm can be used for calculation. The content of Cd in Cd-(GB1)_3_-αMT-(GB1)_3_ fraction was first calculated based on its UV absorbance spectrum (Fig. S3), and the protein concentration was estimated by measuring the absorption at 280 nm. The accurate concentration was measured using the standard DTNB method based on the number of cysteines in the protein (containing DTNB, 8 M urea, and 20 μM EDTA), by Nanodrop 2000 spectrophotometer.

The exact number of zinc or cadmium ions in each protein was analyzed by inductively coupled plasma-mass spectrometry (ICP-MS) at the Center of Modern Analysis, Nanjing University. Optima 5300DV ICP-MS from PerkinElmer was used. The concentration of the Zn was 250 μM, corresponding to 57 μM of αMT (~4.3 eq.) and 54 μM of Zn in 21 μM βMT (~2.6 eq.). The concentration of the Cd was 65 μM, corresponding to 18 μM αMT (~3.6 eq.). All proteins were used immediately after the metal addition and purification for the single-molecule AFM experiment.

**3. Apo-MT and Holo-MT preparation.** The combination of metal-chelating agent EDTA and cysteine-blocking agent NEM (N-ethylmaleimide) was used to eliminate the naturally bound metal in MT. Typically, 10 μL of protein saline solution (0.3 mg/mL) was absorbed onto a glass substrate, then 30 μL of EDTA solution (100 mM, pH 4.5) and 20 μL of NEM aqueous solution (100 mM) were added together and incubated for 20 minutes.

The Zn-form and Cd-form MT proteins were prepared used the Schlenk techniques. The protein was incubated with 20 eq. EDTA (20 mM) for 30 minutes, and then the protein solution was stirred and adjusted pH to 3 slowly using the ammonium formate buffer (20 mM, pH 2.7), which was disposed of with the Chelex 100 resin. Then, the protein was concentrated by Amicon Centrifugal Filters (10K NMWL) to 1 mL and injected into the Schlenk flask, which was evacuated and inflated with the pure N_2_ for three times. Then, the solution was added with the corresponding metal (CdSO_4_, or ZnCl_2_ 600μM, 7.5 eq. for MT, 4.5 eq. for αMT, and 3.5 eq. for βMT) and the reducing agent Tris (2-carboxyethyl) phosphine hydrochloride (TCEP, 30 eq. for each protein, 30 mM). After all these procedures, the pH of the solution was adjusted to 8 slowly using the ammonium formate buffer (20 mM, pH 10) and incubated for 3 hours in the ice bath. Protein was further purified by size-exclusion chromatography (Superdex200 increase 10/300 GL) and anion exchange chromatography (Mono Q 5/50 GL).

**4. Rubredoxin preparation.** Rubredoxin from *Clostridium pasteurianum* was used as a protein with a simple MS_4_ metal center. Fused protein Cys-GB1-Rd-GB1-Cys was used as monomer and then polymerized based on the cysteine for AFM measurement(*1, 2*). Firstly, Rd was denatured using the TCA-βME buffer (10% TCA and 0.5 M β-mercaptoethanol). Then the turbid liquid was centrifuged, and the supernatant was cast away. The operation was repeated three times. At last, the Zn^2+^ or Cd^2+^ was mixed with the apo-Rd. And the mixture was resolved in the βME buffer (0.5 M Tris, 60 mM β-mercaptoethanol, pH 8.0)(*3, 4*).

**5. UV Absorbance of MT polyproteins.** After purification, the concentration of the sample was adjusted to ~10 μM using the centrifugal filters. And the UV absorbance was detected for the Zn-MT, Zn-αMT, Cd-αMT and Zn-βMT by Nanodrop 2000 spectrophotometer (Fig. S2). The UV absorbance for the Cd-form proteins at 250 nm and 280 nm, for the Zn-form proteins at 280 nm was recorded, respectively.

**6. Single-molecule AFM unfolding experiment.** Single-molecule AFM experiments were performed using a Nanowizard4 (JPK) atomic force microscopy. AFM cantilever from Bruker Corp. was used. Tip E with a typical spring constant of ~100 pN/nm and tip D with a constant of ~40 pN/nm in the MLCT mode were used. We used the stiffer tip E to check whether the presence and ratio of one-step and two-step unfolding event is depended on the stiffness of the cantilever. Their overall results and performance was similar. Each cantilever was calibrated in the measurement solution using the equipartition theorem for an accurate spring constant. 100 mM Tris buffer of pH 7.4 was used as the AFM measurement buffer.

The protein solution with a proper concentration (~0.4 mg/mL) was first absorbed onto a clean glass coverslip for optimal single-molecule event detection. The protein solution was incubated for ~20 minutes at room temperature (~25 °C) and then subjected to AFM experiments. The tip of the AFM cantilever contacts the protein-deposited glass surface with an indentation force of ~600 pN for hundreds of milliseconds to randomly pick up a single MT polyprotein by nonspecific interaction, and then retracts at a constant velocity, leading to protein unfolding and rupture of the metal cluster. The pulling distance was 400 nm, with a sample rate of 8800 Hz. Finally, the polyprotein was detached from the AFM tip or coverslip. Then, the tip moved to another position to pick up a new molecule and repeated this cycle. Typically, a single molecule is picked up with a ratio of ~0.1%. The pulling speed was 400 nm/s if not specified.

**7. Bell-Evans model to extract the kinetic from loading rate experiment.** For the one-step rupture scenario of the M_4_S_11_ cluster of αMT under mechanical manipulation, we model this non-equilibrium process as an all-or-none two-state process with a force-dependent rate constant *k(F)*, which can be described by the Bell-Evans model(*5, 6*).

$k\left( F \right)=k_{0}exp\left( \frac{F\Delta x_{\beta}}{k_{B}T} \right)$ (1)

*k*(*F*) is the unfolding/rupture rate constant under a stretching force of *F*, *k*_0_ is the unfolding/rupture rate constant at zero force, Δx_β_ is the distance between the bonded state and the transition state. *k_B_* is the Boltzmann constant, T is the absolute temperature.

Using the dynamic force spectroscopy mode, the slope $a$ of the force−extension curves immediately (~3 nm) before the rupture event was first determined to obtain the loading rate ($r=av$, where $v$ is the pulling speed)(*7*). All the data were fitted with the Bell-Evans model (1), thus yielding the spontaneous rupture rate, and the distance from the bound state to the transition state with the following equation:

$F=\frac{k_{B}T}{\Delta x_{\beta}}ln\left( \frac{\Delta x_{\beta}}{k_{0}k_{B}T} \right)+\frac{k_{B}T}{\Delta x_{\beta}}ln\left( r \right)$ (2)

By performing the single-molecule AFM unfolding experiment under five different pulling speeds, 200 nm/s, 400 nm/s, 1000 nm/s, 2000 nm/s, and 4000 nm/s, the relationship between loading rate and most probable rupture force can be obtained on a log scale (Fig. 6), which is fitted by a linear line as equation (2). Thus, the slope of this line can be used to calculate the Δx_β_, which is the distance between the bonded state and the transition state, and the y-intercept is used to calculate the *k*_0._

**8. Molecular dynamics simulation.** Our multiscale modelling started with the structure of Cd-bound α-subunit MT-III determined by solution NMR (PDB ID 2f5h, chain A). Classical molecular dynamics (MD) simulations were carried out with a 2 fs time-step for 75 ns at 300K using the program GROMACS 4.6.7(*8*) and the CHARMM27 forcefield for protein energetics(*9*). Parameters for Cd-thiolate bonds, angles and dihedrals were copied from Fe-thiolate bonds(14), except that the Cd-S bond equilibrium distance was adjusted to 0.275 nm and the Cd partial charge was set to 0.625 *e*, both estimated from quantum chemical calculations on tetrahedral Cd(SCH_3_)_4_^2-^. Lennard-Jones parameters for Cd were copied from Zn(*9*). Note these are approximate parameters chosen to preserve metal cluster geometry during classical MD. Lys, Glu and Asp side-chains were treated in their charged states, and all Cys side-chains were treated as thiolates bound to Cd ions. Solvation was treated with a Generalized-Born model using the Still method(*8*).

Hybrid QM/MM calculations were performed in various frames taken along this MD trajectory, with the side-chains of all eleven Cys and the four metals in the QM region (total of 59 quantum atoms). The remaining protein was treated in the MM region using the CHARMM27 forcefield. The QM region was modeled with density functional theory (DFT), the M06L functional(*10*) and the def2-SVP basis set(*11*), with a total charge of -3 and singlet spin multiplicity, as all metal centers have a closed-shell. An effective core potential was used for Cd and a second-order procedure was activated to help SCF convergence. Water solvation was treated with the conductor-polarizable continuum model (CPCM). Capped covalent bonds were treated with the link-atom approach. All structural models simulated with the QM/MM potential (either taken from the PDB or the classical MD trajectory) were initially submitted to full geometry optimizations to find energy minima with conjugated gradient and FIRE methods (both within a 1.5 kJ/mol/angstrom RMS gradient tolerance)(*12*). Zn-αMT models were built by exchanging the Cd centers on the respective structure followed by full geometry optimization. Bond-rupture profiles (Fig. 4 in the main text) were determined with constrained geometries simulate external force (COGEF) procedure(*13, 14*) by scanning the C_α_ distance between terminal residues (Lys-32 and Gln-68) as in the AFM experiment. Note that by construction, the COGEF simulations do not allow full protein relaxation and are carried out to a maximum stretch of ~1.2 nm. Thus, we cannot mimic the complete AFM sawtooth-like curves and force-relaxation after M-S bond rupture. Nudge elastic band (NEB) algorithm was used to find addition reaction pathways (Fig. 5B/C). Langevin molecular dynamics at 300K and 1fs time steps were conducted with the hybrid potential (Fig. 5A). All hybrid potential simulations used full electrostatic embedding within the pDynamo 1.9 library(*12*) interfaced to the ORCA 4.1.1 program(*15*) for electronic structure calculations.

**Supplementary Notes**

**1.The amino acid sequence of each protein.**

**Human MT III (MT):**

MDPETCPCPSGGSCTCADSCKCEGCKCTSCKKSCCSCCPAECEKCAKDCVCKGGEAAEAEAEKCSCCQ

**(GB1)_3_-MT-(GB1)_3_:**

M-RGS-HHHHHH-GS- MDTYKLILNGKTLKGETTTEAVDAATAEKVFKQYANDNGVDGEWTYDDATKTFTVTE-RS-

MDTYKLILNGKTLKGETTTEAVDAATAEKVFKQYANDNGVDGEWTYDDATKTFTVTE-RS-

MDTYKLILNGKTLKGETTTEAVDAATAEKVFKQYANDNGVDGEWTYDDATKTFTVTE-RS-

**MDPETCPCPSGGSCTCADSCKCEGCKCTSCKKSCCSCCPAECEKCAKDCVCKGGEAAEAEAEKCSCCQ**-RS-

MDTYKLILNGKTLKGETTTEAVDAATAEKVFKQYANDNGVDGEWTYDDATKTFTVTE-RS-

MDTYKLILNGKTLKGETTTEAVDAATAEKVFKQYANDNGVDGEWTYDDATKTFTVTE-RS-

MDTYKLILNGKTLKGETTTEAVDAATAEKVFKQYANDNGVDGEWTYDDATKTFTVTE

**(GB1)_3_-αMT-(GB1)_3_:**

M-RGS-HHHHHH-GS-

MDTYKLILNGKTLKGETTTEAVDAATAEKVFKQYANDNGVDGEWTYDDATKTFTVTE-RS-

MDTYKLILNGKTLKGETTTEAVDAATAEKVFKQYANDNGVDGEWTYDDATKTFTVTE-RS-

MDTYKLILNGKTLKGETTTEAVDAATAEKVFKQYANDNGVDGEWTYDDATKTFTVTE-RS-

**KSCCSCCPAECEKCAKDCVCKGGEAAEAEAEKCSCCQ**-RS-

MDTYKLILNGKTLKGETTTEAVDAATAEKVFKQYANDNGVDGEWTYDDATKTFTVTE-RS-

MDTYKLILNGKTLKGETTTEAVDAATAEKVFKQYANDNGVDGEWTYDDATKTFTVTE-RS-

MDTYKLILNGKTLKGETTTEAVDAATAEKVFKQYANDNGVDGEWTYDDATKTFTVTE

**(GB1)_3_-βMT-(GB1)_3_:**

M-RGS-HHHHHH-GS-

MDTYKLILNGKTLKGETTTEAVDAATAEKVFKQYANDNGVDGEWTYDDATKTFTVTE-RS-

MDTYKLILNGKTLKGETTTEAVDAATAEKVFKQYANDNGVDGEWTYDDATKTFTVTE-RS-

MDTYKLILNGKTLKGETTTEAVDAATAEKVFKQYANDNGVDGEWTYDDATKTFTVTE-RS-

**MDPETCPCPSGGSCTCADSCKCEGCKCTSCK**-RS-

MDTYKLILNGKTLKGETTTEAVDAATAEKVFKQYANDNGVDGEWTYDDATKTFTVTE-RS-

MDTYKLILNGKTLKGETTTEAVDAATAEKVFKQYANDNGVDGEWTYDDATKTFTVTE-RS-

MDTYKLILNGKTLKGETTTEAVDAATAEKVFKQYANDNGVDGEWTYDDATKTFTVTE

**Rubredoxin from *Clostridium pasteurianum* (Rd):**

MKKYTCTVCGYIYNPEDGDPDNGVNPGTDFKDIPDDWVCPLCGVGKDQFEEVEE

**Cys-GB1-Rd-GB1-Cys**

M-RGS-HHHHHH-GS-

CMDTYKLILNGKTLKGETTTEAVDAATAEKVFKQYANDNGVDGEWTYDDATKTFTVTERSM**KKYTCTVCGYIYNPEDGDPDNGVNPGTDFKDIPDDWVCPLCGVGKDQFEEVEE**RSMDTYKLILNGKTLKGETTTEAVDAATAEKVFKQYANDNGVDGEWTYDDATKTFTVTERSC

**2. AFM data selection criteria and data analysis procedure.**

Force-extension curves are first screened automatically by JPK Data Processing program to eliminate featureless curves without picking up any molecule, and then they are further selected and analyzed manually using a custom-written program in Igor Pro 6. The curves are analyzed by fitting the polymer elasticity using the Worm-like chain (WLC) model leading to the contour length for each peak by the following equation

$F(\mathrm{x})=\frac{k_{B}T}{P}\left[\frac{1}{4}\left(1-\frac{\mathrm{x}}{Lc}\right)^{-2}-\frac{1}{4}+\frac{\mathrm{x}}{Lc}\right]$ (3)

*F*(x) is the force applied to the polymer (protein backbone) under a polymer extension x. *P* is the persistence length. *Lc* is the contour length. *k*_B_ is the Boltzmann constant, and T is the temperature in Kelvin. The dashed line in the force-extension curve is from this fitting.

And the contour length increment (ΔLc) for each force peak upon specific protein unfolding is obtained. For example, ΔLc of marker protein GB1 is ~18 nm, αMT is ~11 nm, and βMT is ~9 nm. Because MT is sandwiched by three marker proteins on both sides as (marker)_3_-MT-(marker)_3_, we only select the curves from (GB1)_3_-MT-(GB1)_3_ with at least three unfolding peaks from the marker protein GB1 with additional peak(s) for analysis. This criterion ensures the sandwiched MT protein is stretched and measured. For stepwise unfolding pathway of αMT in protein construct (GB1)_3_-αMT-(GB1)_3_, we only select the curve with two unfolding peaks besides the marker protein signal for analysis, and only the sum of the two peaks’ ΔLc equals to ~11 nm was included for the result. A similar procedure was used for βMT in (GB1)_3_-βMT-(GB1)_3_.

**3.Why is single domain MT used for metal cluster characterization ?**

The one-step unfolding event of α and β domain in the full-length MT show single unfolding peak with a ΔLc of 9 nm and 11 nm, which can be distinguished. However, besides the one-step event, stepwise unfolding events were also observed for both domains, which can show peak(s) with similar ΔLc and disable the clear identification of unfolding events for each domain. For example, in Supplementary Figure 5, the 11 nm peak is split to ~2+9 nm while the 9 nm peak is split to ~2+7 nm. For a high standard and clear result, we used single domain MT for all further AFM experiments.

**4.The assignment of two-step unfolding events to four different pathways**

The theoretical ΔLc combination of the two peaks for each two-step rupture pathways of the metal cluster can be first calculated based on the location of the four boundary cysteines in αMT (Table S1). Here, we split the length of the boundary cysteine to both sides as 0.5 aa. Based on this eight number (b), we can assign the data into eight groups as: 10.1; 8.6; 7.5; 5.7; 5.1; 3.6; 2.3 and 1.0 nm. The first principle is that we use b±0.5 as the boundary to classify the data. For example, the ΔLc of one peak of any event is bigger than 9.6 nm (10.1±0.5) or smaller than 1.5 nm (1.0±0.5) will be assigned to the 10.1+1.0 nm pathway. Then, peaks outside of this range, which boundary the ΔLc of the peak is closer to, it is assigned to this group. For example, a peak with ΔLc of 9.3 will still be assigned to the 10.1+1.0 nm pathway. As a result, most data can be classified. Finally, if the assignment of the two stepwise peaks is against each other, such as 9.0+1.0 nm, the event will be assigned to the peak, which is closer to the theoretical boundary like 1.0 nm here and is assigned to the 10.1+1.0 nm pathway.

1. **Boundary cysteine during the stepwise unfolding of βMT is difficult to resolve**

Like αMT, several different two-step unfolding pathways of βMT were observed, showing a broad and continuous ΔLc from 1 nm to 9 nm, further supporting the plastic and dynamic nature of the hexatomic metal cluster in βMT (Fig. S12, *n*=57). Several different two-step rupture pathways were observed. For example, curve 1 shows stepwise unfolding peaks of βMT with a first ΔLc of 5.7 nm and a second ΔLc of 4.2 nm. Curve 2 shows stepwise peaks with a ΔLc of 7.5 nm and a ΔLc of 2.6 nm. However, the X-ray structure of β domain of human MT III has not been solved yet. In addition, its nine metal-coordinating cysteines are closer to each other, leading to a smaller ΔLc difference (<1 nm) for most possible two-step rupture pathways. These factors prevent a clear identification of the boundary cysteines for each pathway.


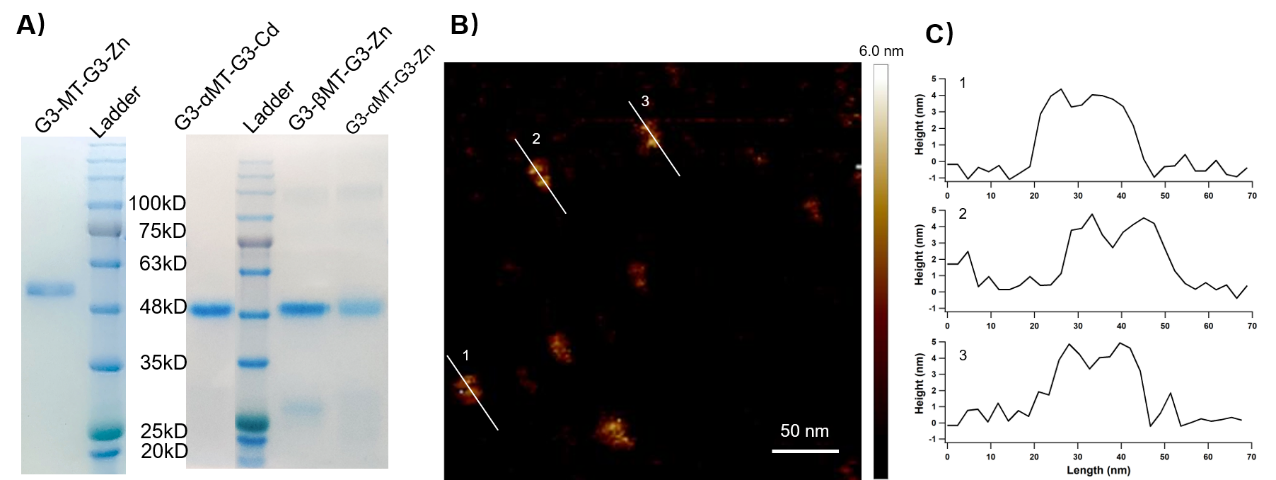
**Supplementary Figures**


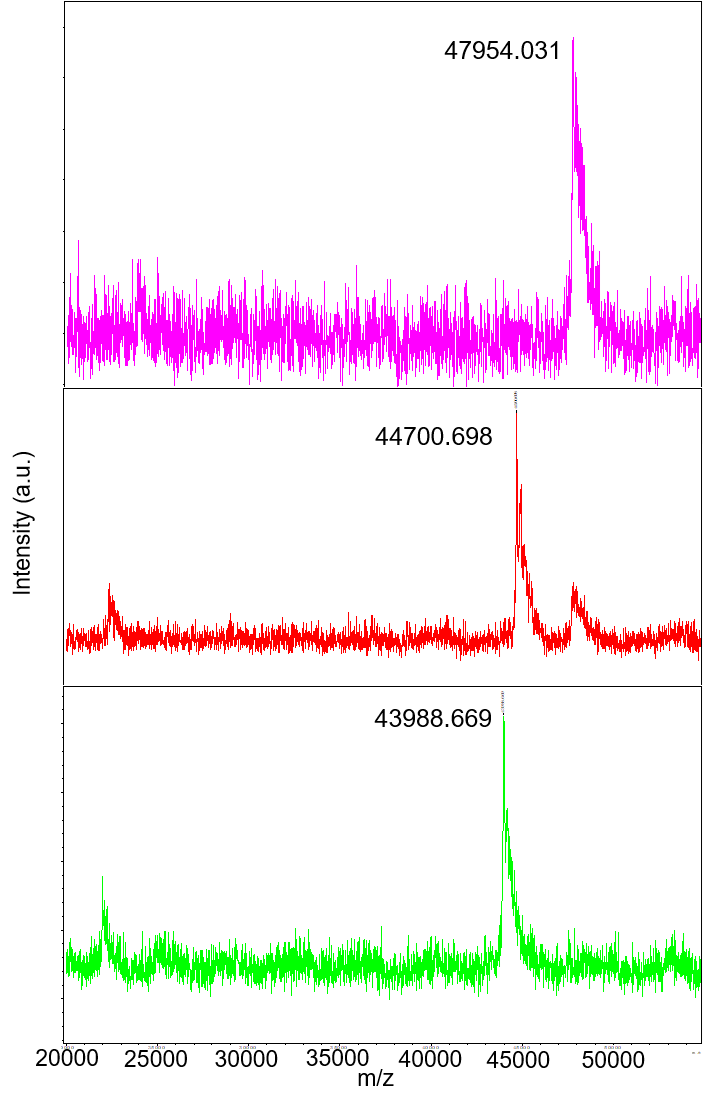
Figure S1. A) SDS-PAGE gel results for MT polyproteins, (GB1)_3_-xMT-(GB1)_3_. The samples from left to right: Zn-MT (~48 kD), Cd-αMT (~45 kD), Zn-βMT (~44 kD) and Zn-αMT (44.684 kD). All samples were prepared with the addition of DTT. B) AFM imaging of (GB1)_3_-Zn-MT-(GB1)_3_. C) The height of three selected polyproteins.

Figure S2. The MALDI-TOF-MS result of Zn-MT polyprotein, (GB1)_3_-xMT-(GB1)_3._ Zn-MT (theoretical MW: 47.980 kD), Zn-αMT (44.673 kD) and Zn-βMT (43.938 kD).


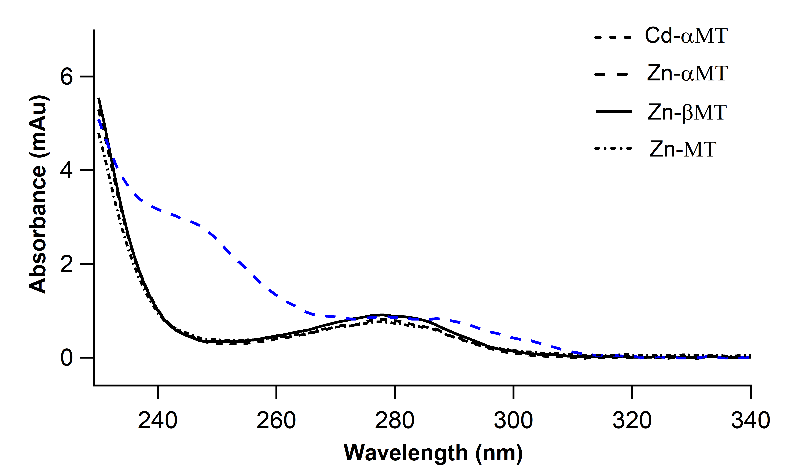


Figure S3. The UV-vis absorbance spectrum of MT polyprotein, (GB1)_3_-xMT-(GB1)_3._ Cd-αMT, Zn−αMT, Zn-βMT and Zn-MT.


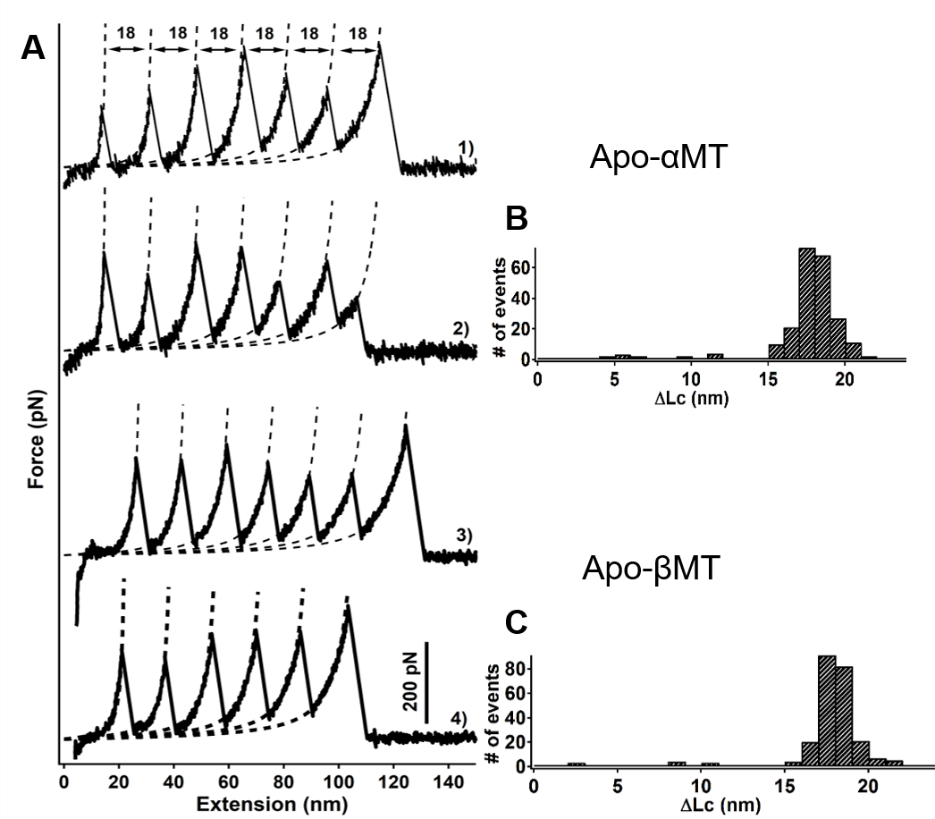


Figure S4. AFM unfolding results of Apo-αMT and Apo-βMT. Representative Force-extension curves of the unfolding of polyprotein Apo-αMT (curves 1 and 2) and Apo-βMT (curves 3 and 4) show only the unfolding signal from marker protein GB1 with ΔLc of 18 nm. The unfolding histogram further demonstrates that most apo-αMT (B) and apo-βMT (C) do not show rupture peaks compared to their holo-form.


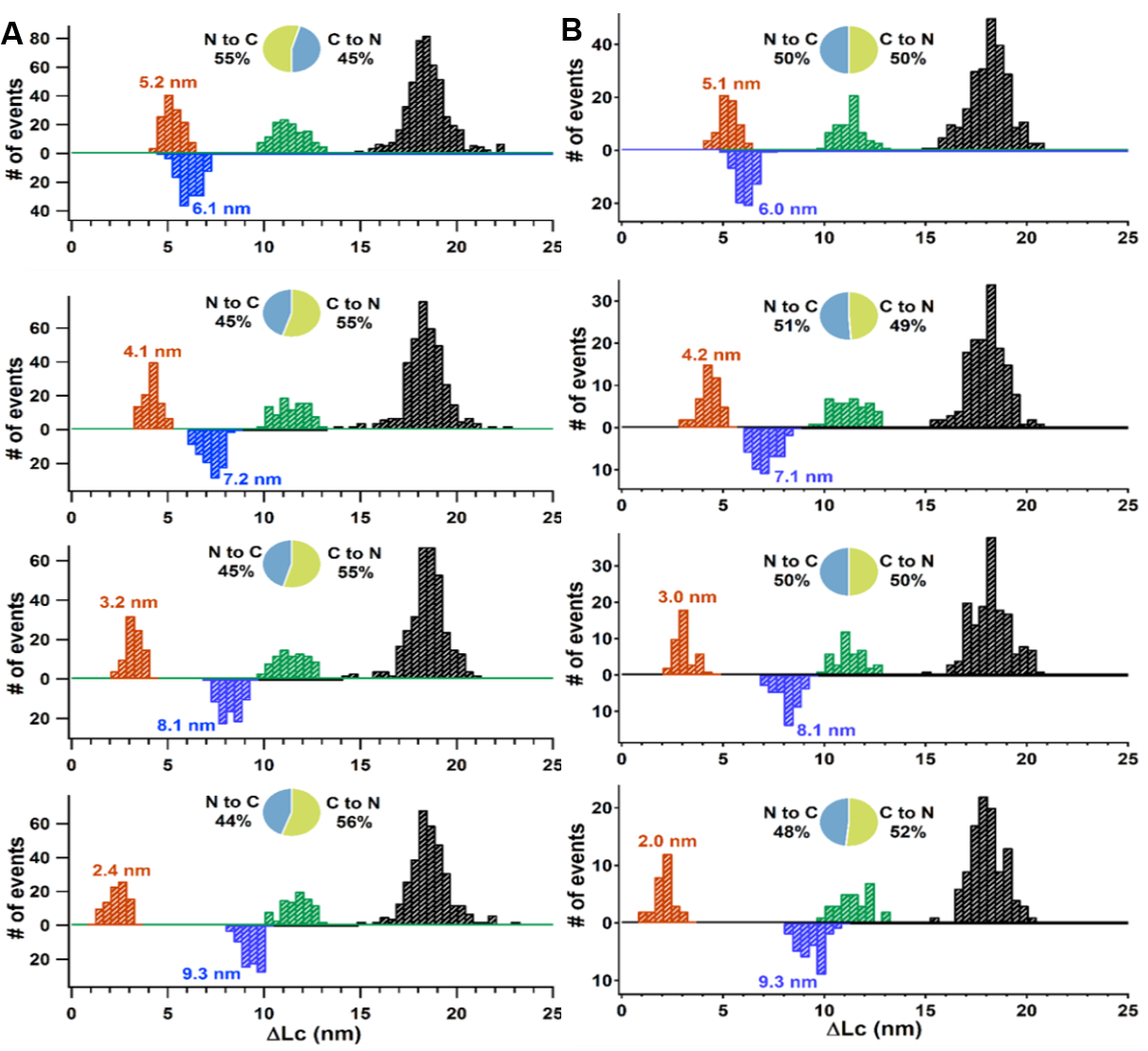
Figure S5. ΔLc histogram of unfolding/rupture peaks in the two-step rupture scenarios of Zn-αMT (A) and Cd-αMT (B). These diagrams are supplementary to Figure 3 in the main text and Supplementary Figure 10. Here the cumulative ΔLc (~11 nm, in green) of the two peaks from stepwise α/βMT unfolding as well as the ΔLc of fingerprint GB1 unfolding (~18 nm, in gray) are shown. A pie chart in each graph describes the probability of unfolding direction from two ends.


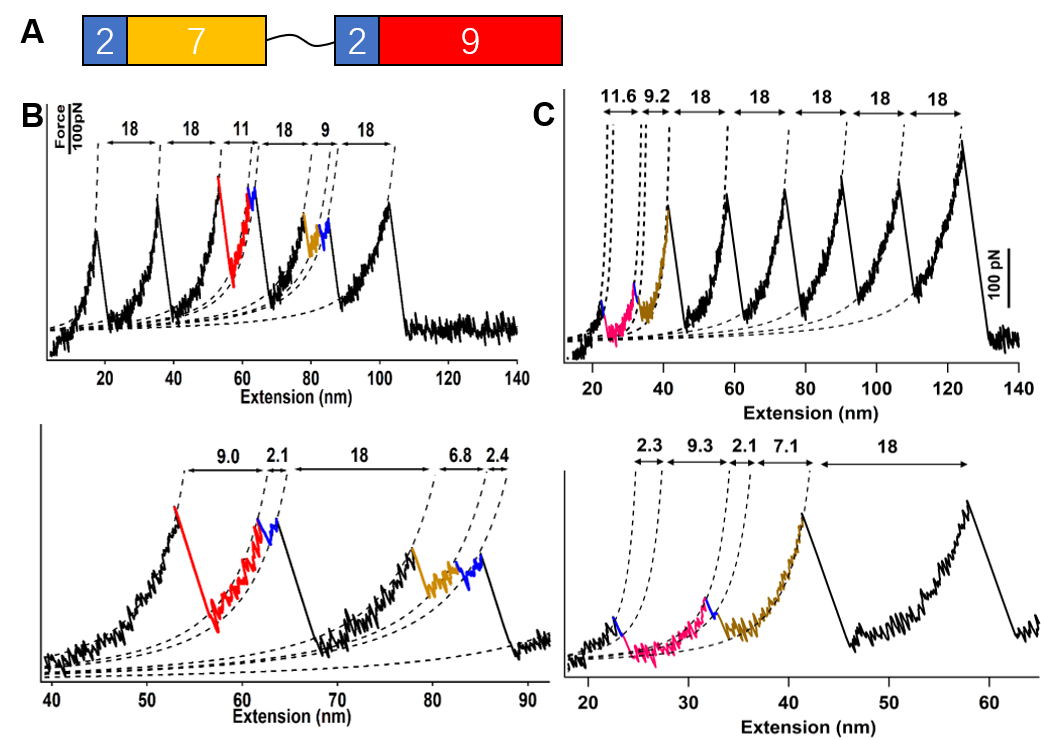


Figure S6. Force-extension curves of stepwise ruptured (GB1)_3_-MT-(GB1)_3_ showing peaks with similar ΔLc value from two domains. A) an α domain showed a ~2+7 nm unfolding pathway and a β domain showed a ~2+9 nm pathway. Thus, two peaks with ~2 nm will be observed and difficult to be assigned to a specific domain unambiguously. B-C) Two force-extension curves of full-length MT unfolding show such a phenomenon. The rupture peaks of MTare enlarged at the bottom graph for clarity.


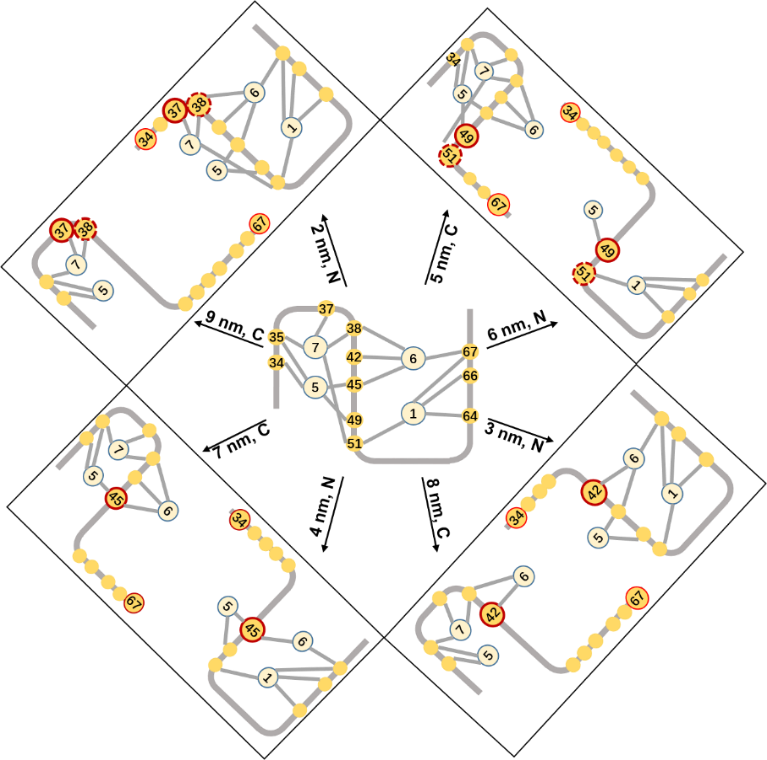


Figure S7. Possible 2D schematics of the rupture mechanism of the M_4_S_11_ cluster. The M_4_S_11_ cluster in the folded αMT is shown in the center, highlighting all metal-bound cysteines. The eight partially ruptured metal cluster intermediates among different unfolding pathways are shown. The boundary cysteine of the intermediate is marked by a red circle. Except for the boundary M-S bond, it is unknown whether other M-S bonds are still bound and are shown here only for a better understanding of the intermediate.


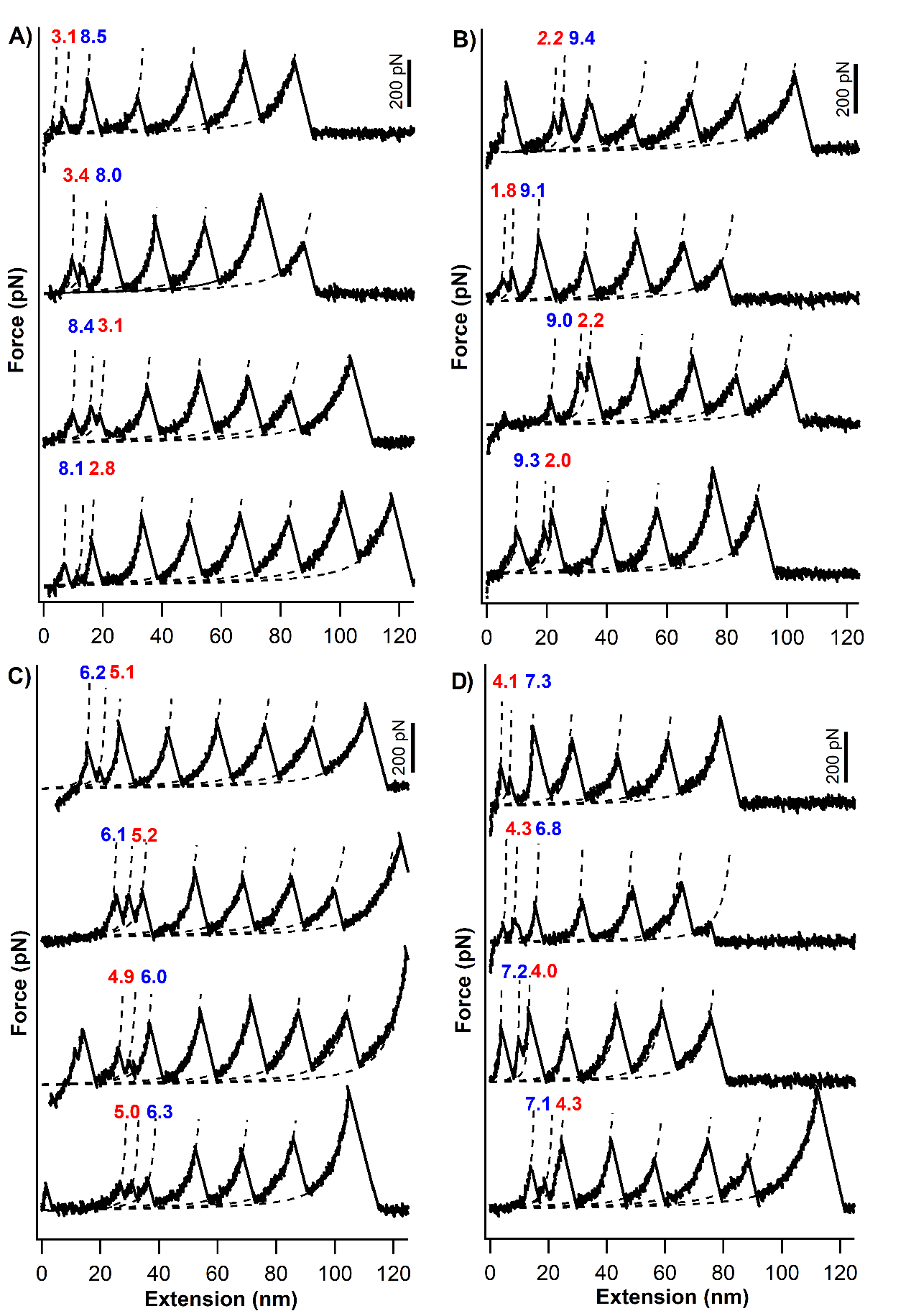


Figure S8. More representative unfolding curves of the four different two-step rupture pathways of the Zn_4_S_11_ cluster in αMT detected by AFM-SMFS, including 3.2+8.1 nm A); 2.3+9.3 nm B); 5.2+6.1 nm C) and 4.1+ 7.2 nm D), unfolding from both termini.


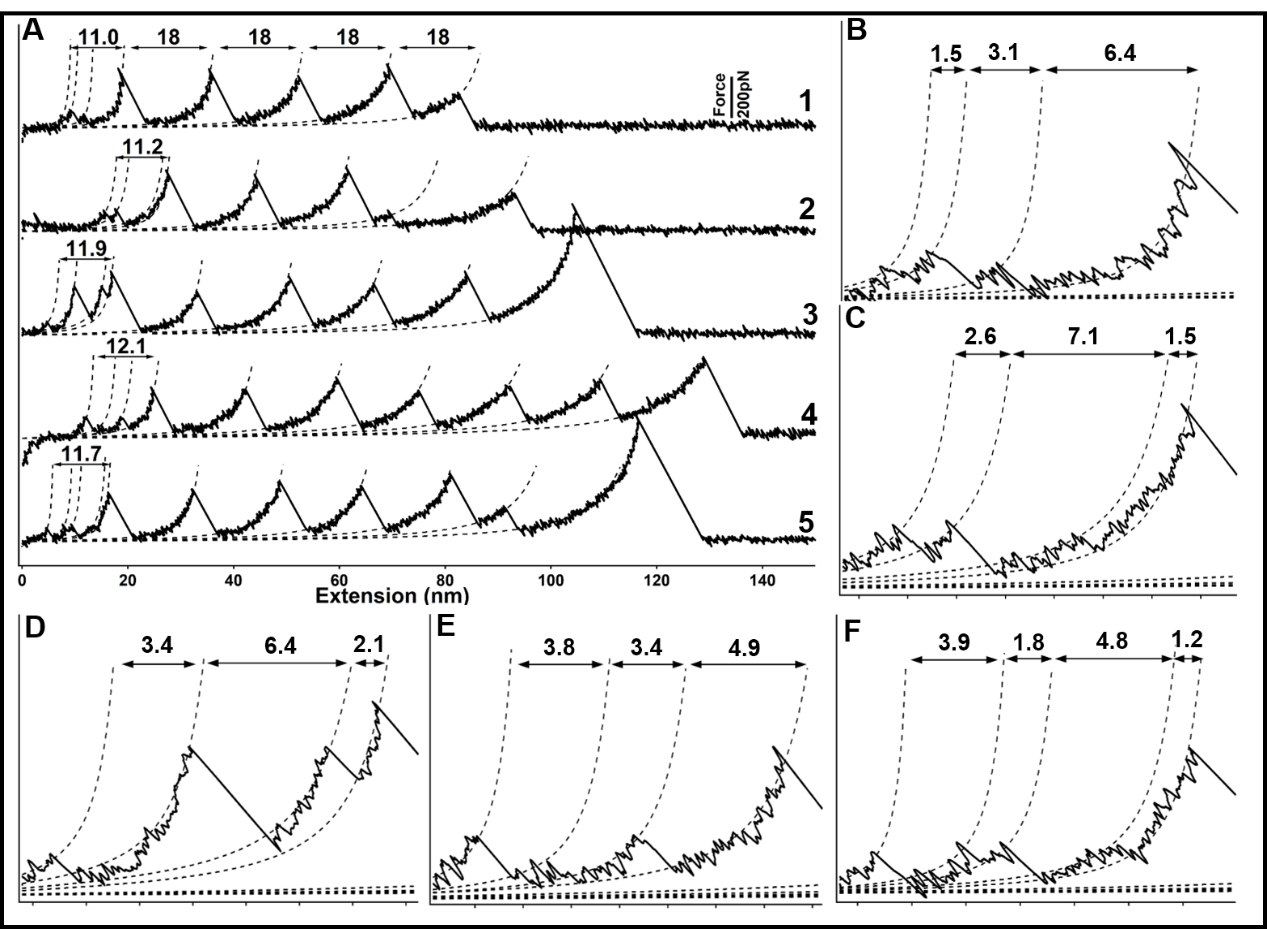
Figure S9. Force-extension curves of the multi-step rupture scenario of the Zn_4_S_11_ in αMT. A) Five multi-step curves were found among ~800 αMT unfolding curves (<1%). The peaks for the cluster rupture are enlarged for clarity, as five different combinations: B) 1.5+3.1+6.4 nm; C) 2.6+7.1+1.5 nm; D) 3.4+6.4+2.1nm; E) 3.8+3.4 +4.9 nm and F) 3.9+1.8+4.8 +1.2 nm.


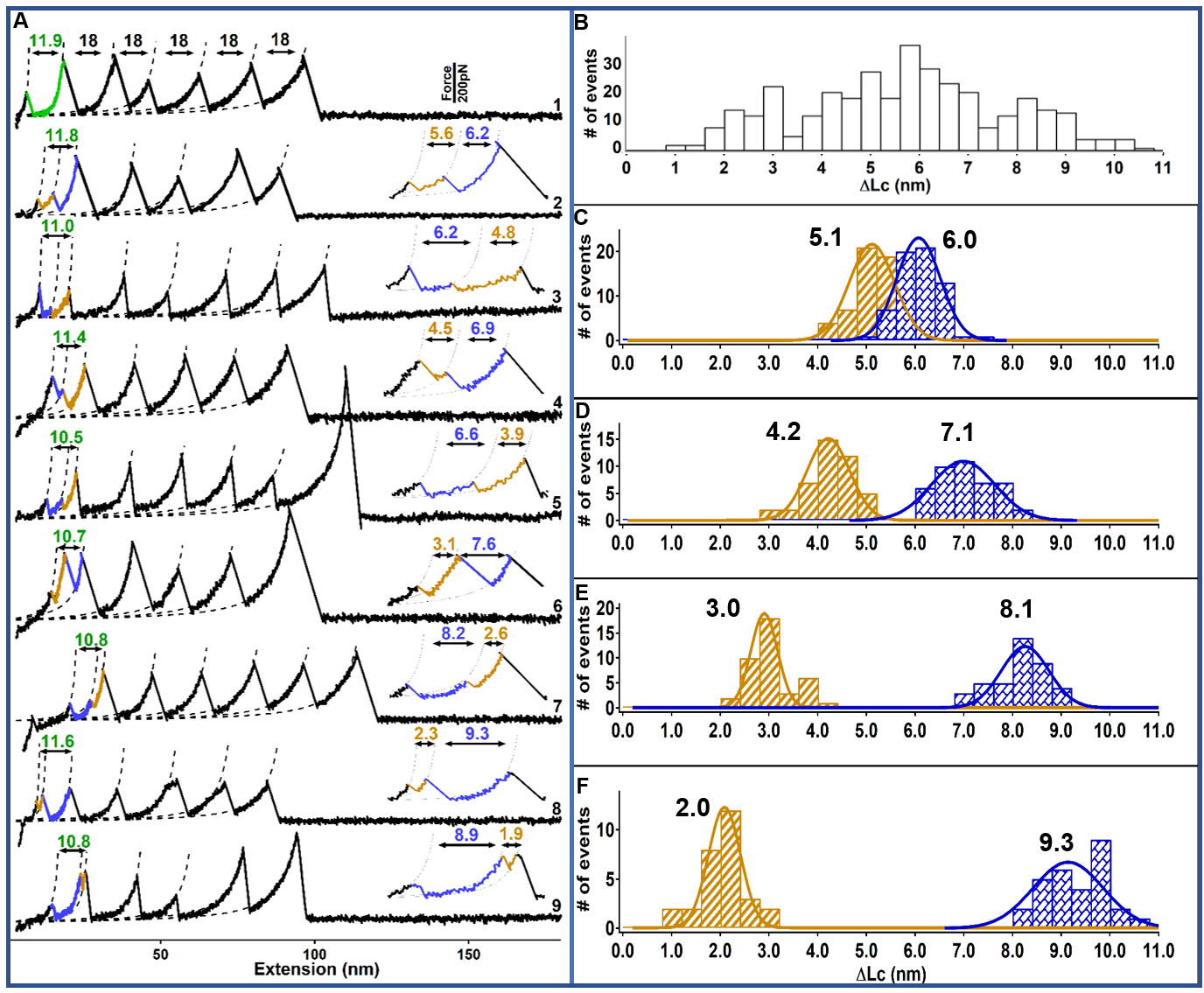


Figure S10. AFM unfolding experiment results of Cd-αMT. A) Representative force-extension curves of (GB1)_3_-Cd-αMT-(GB1)_3_ show the one-step (curve 1) and multiple two-step rupture patterns of Cd-αMT (curve 2-9). B) ΔLc histogram of two-step unfolding scenario of Cd-αMT shows a broad and continuous distribution. C-F) ΔLc histogram for each two-step rupture event shows similar four pathways as Zn-αMT. The bin size is 0.4 nm.


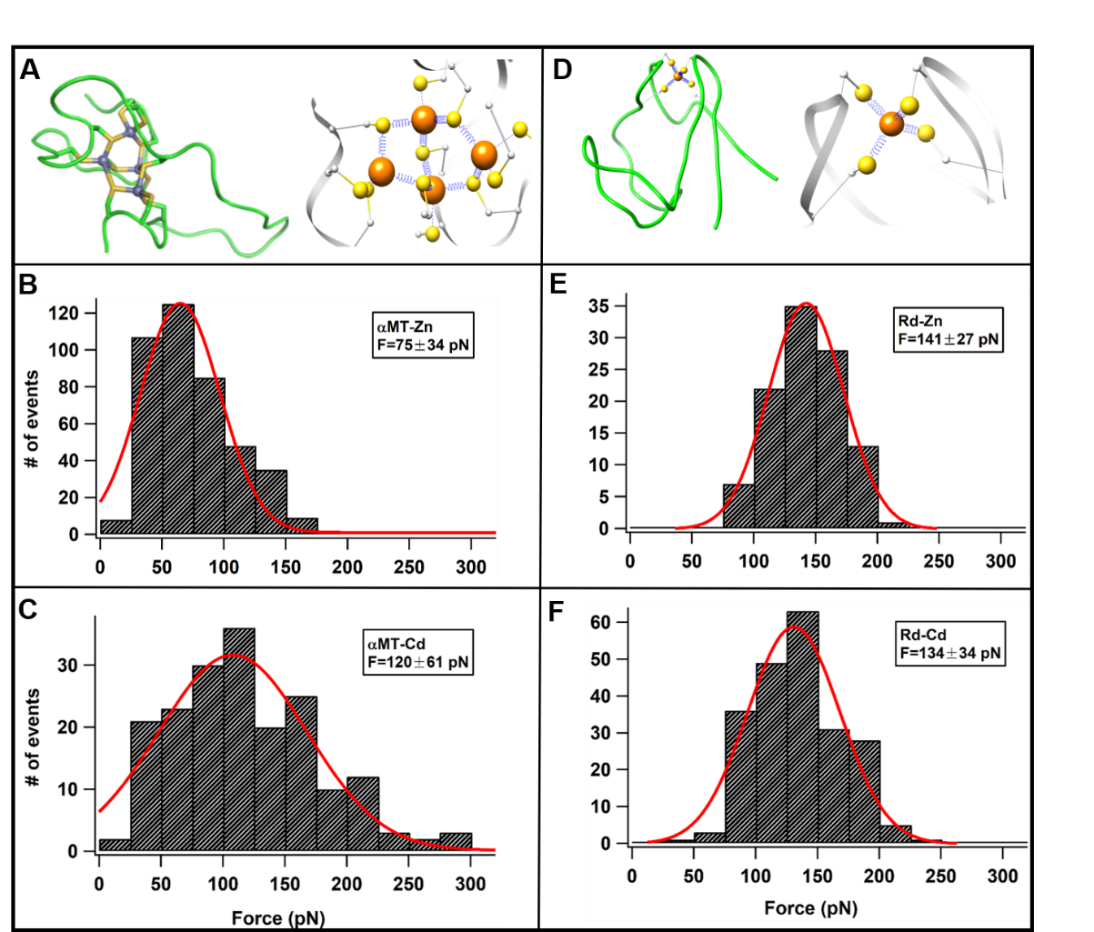


Figure S11. Rupture force histogram of one-step unfolding of Zn/Cd-αMT and Rd. Scheme of αMT (A) and Rd (B). The protein structure is shown on the left, and an enlarged graph of the metal center is shown on the right. The metal is colored in brown and sulfur is colored in yellow. The average rupture force is 75±34 pN for Zn-αMT (B); 120±61 pN for Cd-αMT (C); 141±27 pN for Zn-Rd (E); and 134±34 pN for Cd-Rd (F).


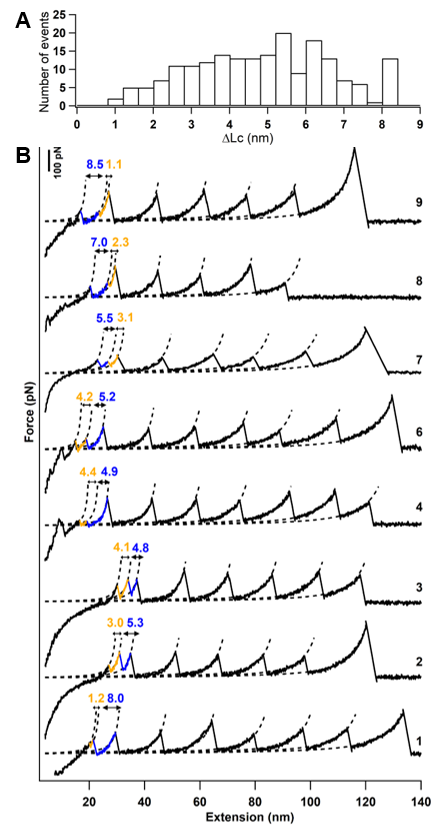


Figure S12. AFM unfolding experiment results of Zn-βMT. A) ΔLc histogram of two-step unfolding of Zn_3_S_9_ cluster in Zn-βMT show a wide and continuous distribution similar as the Zn_4_S_11_ cluster in Zn-αMT but from 1 nm to 8.5 nm. The bin size is 0.4 nm. B) Representative force-extension curves of the two-step unfolding of Zn-βMT show several different pathways.

**Supplementary Table**

Table S1. Theoretical ΔLc for the four two-step rupture scenarios of αMT

| ΔLc combination | Cysteine rupture pattern | Theoretical ΔLc for the first peak | Theoretical ΔLc for the second peak |
| --- | --- | --- | --- |
| 6.1+5.2 | C67-C49-C34 | 18.5*0.36-0.97=5.7 | 15.5*0.36-0.43=5.1 |
| 7.2+4.1 | C67-C45-C34 | 22.5*0.36-0.65=7.5 | 11.5*0.36-0.54=3.6 |
| 8.1+3.2 | C67-C42-C34 | 25.5*0.36-0.58=8.6 | 8.5*0.36-0.75=2.3 |
| 9.3+2.3 | C67-C38-C34 | 29.5*0.36-0.57=10.1 | 4.5*0.36-0.66=1.0 |

**Supplementary Video:** Animation of the molecular dynamics simulation presented in Fig. 5A, using the same color code for M-S bonds. Distances in angstroms and two viewing angles are shown. Protein backbone and Cys side-chains are in green tubes, with sulfur in yellow and zinc in gray.

**Supplementary References:**

1. P. Zheng, Y. Cao, H. Li, *Langmuir* **27**, 5713-5718 (2011).

2. P. Zheng, H. Li, *J. Am. Chem. Soc.* **133**, 6791-6798 (2011).

3. Z. Dauter, K. S. Wilson, L. C. Sieker, J. M. Moulis, J. Meyer, *Proc. Natl. Acad. Sci. U. S. A.* **93**, 8836-8840 (1996).

4. Z. G. Xiao *et al.*, *J. Am. Chem. Soc.* **120**, 4135-4150 (1998).

5. E. Evans, K. Ritchie, *Biophys. J.* **72**, 1541-1555 (1997).

6. E. Evans, *Annu. Rev. Biophy. Biomol. Struct.* **30**, 105-128 (2001).

7. K. Klamecka, P. M. Severin, L. F. Milles, H. E. Gaub, H. Leonhardt, *Phys. Biol.* **12**, (2015).

8. B. Hess, C. Kutzner, D. van der Spoel, E. Lindahl, GROMACS 4: *J. Chem. Theory. Comput.* **4**, 435-447 (2008).

9. A. D. MacKerell, M. Feig, C. L. Brooks, *J. Am. Chem. Soc.* **126**, 698-699 (2004).

10. Y. Zhao, D. G. Truhlar, *J. Chem. Phys.* **125**, (2006).

11. F. Weigend, R. Ahlrichs, *Phys. Chem. Chem. Phys.* **7**, 3297-3305 (2005).

12. M. J. Field, *J. Chem. Theory. Comput.* **4**, 1151-1161 (2008).

13. M. K. Beyer, *J. Chem. Phys.* **112**, 7307-7312 (2000).

14. P. Zheng, G. M. Arantes, M. J. Field, H. Li, *Nat. Commun.* **6**, 7569 (2015).

15. F. Neese, *Wires. Comput. Mol. Sci.* **2**, 73-78 (2012).
